# A new insight in chimeric antigen receptor-engineered T cells for cancer immunotherapy

**DOI:** 10.1186/s13045-016-0379-6

**Published:** 2017-01-03

**Authors:** Erhao Zhang, Hanmei Xu

**Affiliations:** 1 The Engineering Research Center of Peptide Drug Discovery and Development, China Pharmaceutical University, Nanjing, 210009 China; 2 State Key Laboratory of Natural Medicines, Ministry of Education, China Pharmaceutical University, Nanjing, 210009 China

**Keywords:** Adoptive cell therapy, Adverse effects, Switchable dual-receptor T cell, Bifunctional molecule, Immune checkpoint blockade

## Abstract

Adoptive cell therapy using chimeric antigen receptor (CAR)-engineered T cells has emerged as a very promising approach to combating cancer. Despite its ability to eliminate tumors shown in some clinical trials, CAR-T cell therapy involves some significant safety challenges, such as cytokine release syndrome (CRS) and *“on-target, off-tumor”* toxicity, which is related to poor control of the dose, location, and timing of T cell activity. In the past few years, some strategies to avoid the side effects of CAR-T cell therapy have been reported, including suicide gene, inhibitory CAR, dual-antigen receptor, and the use of exogenous molecules as switches to control the CAR-T cell functions. Because of the advances of the CAR paradigm and other forms of cancer immunotherapy, the most effective means of defeating the cancer has become the integration therapy with the combinatorial control system of switchable dual-receptor CAR-T cell and immune checkpoint blockade.

## Background

A specific type of immune therapy, known as a green therapy, has emerged as an exciting new approach for cancer therapy, especially the adoptive cells transfer (ACT) therapy [1–3]. Unlike surgery, radiotherapy, and chemotherapy, the cell-based therapy can facilitate accurate decisions and execute highly complex behaviors [4–6]. The autologous lymphocytes isolated from a patient’s own peripheral blood are endowed with the ability of tumor antigen specificity and rendered capable of eliminating cancer cells expanded ex vivo, and then reinfused into the patient to attack any malignant tumor [7–10]. The majority of preclinical and clinical data regarding autologous T cells have highlighted the safety of using the cell therapy and the lack of potential for graft-versus-host disease (GvHD) mediated by the allogeneic T cells [11–13]. The T cell receptor (TCR), an α/β heterodimer, has the ability to redirect the tumor antigens in a major histocompatibility-complex-dependent (MHC) manner [14–16]. The conventional T cells are activated upon TCR combining with other cell surface molecules, termed TCR/CD3 complex, which contains 10 immunoreceptor tyrosine activation motifs (ITAMs) and 20 tyrosine-phosphorylation sites [17–19]. The tumor escape mechanism, however, is associated with the downregulation of the MHC molecule on the surface of the tumor cell, which restrains the homing of T cells because the interaction between T cell receptor and peptide-MHC is a prerequisite for T cell activation [20–22]. In contrast, the structure and signaling pathway of the chimeric antigen receptor (CAR) are delicate. CARs permanently endow the patient-derived T cells with the ability to recognize and kill any tumor cells expressing the antigens without MHC molecules, and render the tumor cell *“visible”* to T cell immune surveillance [23–25]. Recent advances in genetic engineering and improved recognition of T cells have resulted in the design of new receptor mechanisms, termed CARs. Human T cells modified with this synthetic receptor can specifically redirect tumor antigens and undertake the striking efficacy for many human malignancies [26–28].

**Fig. 1:**
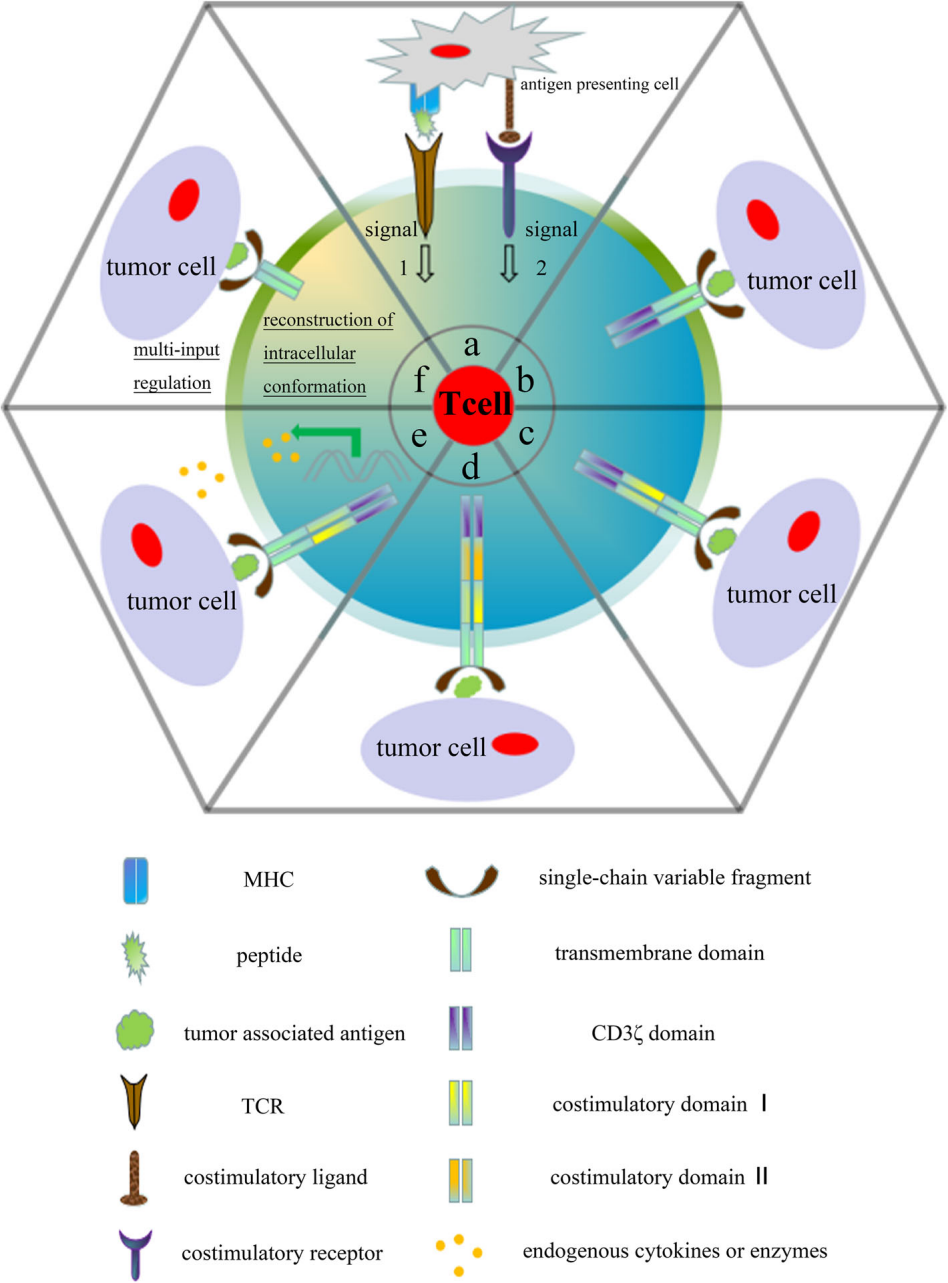
Schematic diagram of TCR- and CAR-modified T cells in adoptive T cells therapy. **a** Activation, proliferation, and cytotoxicity of the T cell are dependent upon the dual signal pathway that includes the T cell receptors (TCRs) that recognize peptide antigens which were processed by the antigen-presenting cells and presented upon the major histocompatibility complex (MHC) of a target cells, and the costimulatory receptor of T cell simultaneously engages a ligand, such as CD28 and B7 molecules. **b** The first-generation CAR contains only the antigen recognition signal, CD3ζ domain, resulting in the transient activation and proliferation of the CAR-T cell based on scFv specificity. **c**–**d** The second- and third-generation CARs contain one and two additional costimulatory signaling domains, respectively, such as CD28, CD137 (4-1BB), and CD134 (OX40). The costimulatory signaling domains can facilitate greater proliferation of modified-T cell and greater cytotoxicity than first-generation CAR. **e** To significantly enhance the overall cytotoxicity of the modified-T cell, the fourth-generation CAR-T cell is generally modified to express CARs with an inducible cytokine genes, such as IL-12 or heparinase, which can stimulate T cell to reach the surface of tumor cells in degrading the extracellular matrix (ECM) within the tumor microenvironment and blocking the inhibitory signaling pathway. **f** The next-generation structure of the CARs with effective specificity for target cells lacking several side effects to the body will be generated in the near future, including reconstruction of endogenous structure and introduction of exogenous regulatory

Like that of the conventional T cell, the structure of CAR-modified T cell contains three moieties, i.e., an extracellular domain, single-chain antibody fragments (scFv), that recognize and bind a specific tumor antigen independent of MHC molecule, a transmembrane domain that usually comprises the homodimer of CD3 or CD8 molecule, and an intracellular signaling domain including a signal-transduction component of the T-cell receptor (e.g., CD3ζ or FcεRIγ) and a costimulatory receptor (e.g., 4-1BB, CD28, or OX40) (Fig. 1) [29–32]. The initial CAR-T cell comprises the scFv element and the CD3ζ signaling domain, which endows the T cell with the abilities of homing and activation (Fig. 1b). However, the cytotoxicity of first-generation CAR-T cells is transient in vivo. To enhance the durability of CAR-T cell cytotoxicity, the second- and third-generation CAR were developed by addition of single and dual costimulatory signaling domains respectively (Fig. 1c, d) [33, 34]. During the last decade, CAR-T cells have shown impressive results in patients with hematological tumor, but have limitations in treating solid tumors probably due to the blunt immune-surveillance that the immune suppressor cells, cytokines, and some proteins hinder T cell functions in tumor microenvironment [35–39]. To overcome this weakness, Koneru et al. recently developed a new module of CAR-T cells simultaneously transduced with both CAR and IL-12 genes, known as armored CAR-T cells, which can penetrate the ovarian tumor site with surmounting the tumor microenvironment [40–42]. Some researchers have also demonstrated that the release of specific enzymes by T cells, known as heparanase (HPSE), which can help immunocytes pass through physical barriers with degradation of extracellular matrix (ECM) that possesses an ability to prevent the T cells homing to tumor site. Some chemokine receptors have also been introduced into CAR-T cell, which can drive effective T cell infiltration into the tumor bed (Fig. 1e) [43–45].

Despite the promising clinical results, CAR-T cell therapy also involves several deleterious types of toxicity due to the inability to control T cell activity and some tumor-associated antigens that are presented by both diseased and healthy tissue. The prominent toxicity of CAR-T cell therapy involves cytokine release syndrome (CRS) and “on-target, off-tumor” toxicity [46–49]. The CRS effect, so-called cytokine-associated toxicity, is a result of intense tumor-killing responses mediated by large numbers of activated lymphocytes (B cells, T cells, and natural killer cells) [50]. According to a previous clinical trial, NCT0265014, the levels of several cytokines including interleukin 6 (IL-6) and interferon γ (IFN-γ) are markedly elevated in patient serum after receiving genetically engineered T cells. At the early stage of CAR-T cell therapy, large numbers of CAR-T cells are reinfused into patients with refractory and relapse malignancies. This results in rapid elimination of tumor cell and extends the patient survival considerably [51, 52]. Concomitantly, the obtained cytokine levels on patients with the administration of adoptive T-cell therapy are several hundred times higher than the baseline level, which typically causes a clinical syndrome including fever, hypotension, and neurological changes, potentially leading to rapid death [53, 54]. Lee et al. published a grading system for assessing the severity of CRS. In this system, the CRS has five grades based on the clinical signs and symptoms [55]. As conventional adverse effects derived from drugs, CRS toxicity can be controlled by reducing the dosage of the active T cells. However, the numbers of T cell are difficult to control and may eventually exceed a threshold, resulting in some severe side responses. In addition to CRS, the second safety concern associated with the CAR-T cell therapy is a normal tissue damage attributable to the presence of the tumor antigens on the peritumoral tissues [56, 57]. In current reports, tumor antigens that are expressed on cancer cells but not on normal cells are rare, especially for solid tumors [58]. In this way, tumor-associated antigens are often currently used as targeting molecules for CARs. The lethal toxicity described as “on-target, off-tumor” effect, to date, has been reported in some cancer immunotherapy clinical trials covering the infusion of engineered-T cells. For instance, the most successful therapy for targeting the B-cell malignancies has been reported after infusion of CAR-T cell with specificity to CD19 in an ongoing clinical trial NCT00924326. Owing to the targeting and eradication of normal B cells, long-term B-cell aplasia symptoms appear in patients with autologous T cell expressing the second-generation CAR with CD19 scFv [59]. This off-tumor expression of the interesting target molecule in normal cell leads to rapid cardiopulmonary toxicity [60]. For another case, a patient with the metastatic renal cell carcinoma expressing carboxyanhydrase-IX (CAIX) obtains the common toxicity criteria (CTC) grade 2–4 liver enzyme disturbances after receiving the CAR-modified T cell against CAIX antigen [61]. All of these types of on-target toxicity generally stem from the inability of CAR-T cell to distinguish between normal cell and cancer cell.

**Fig. 2:**
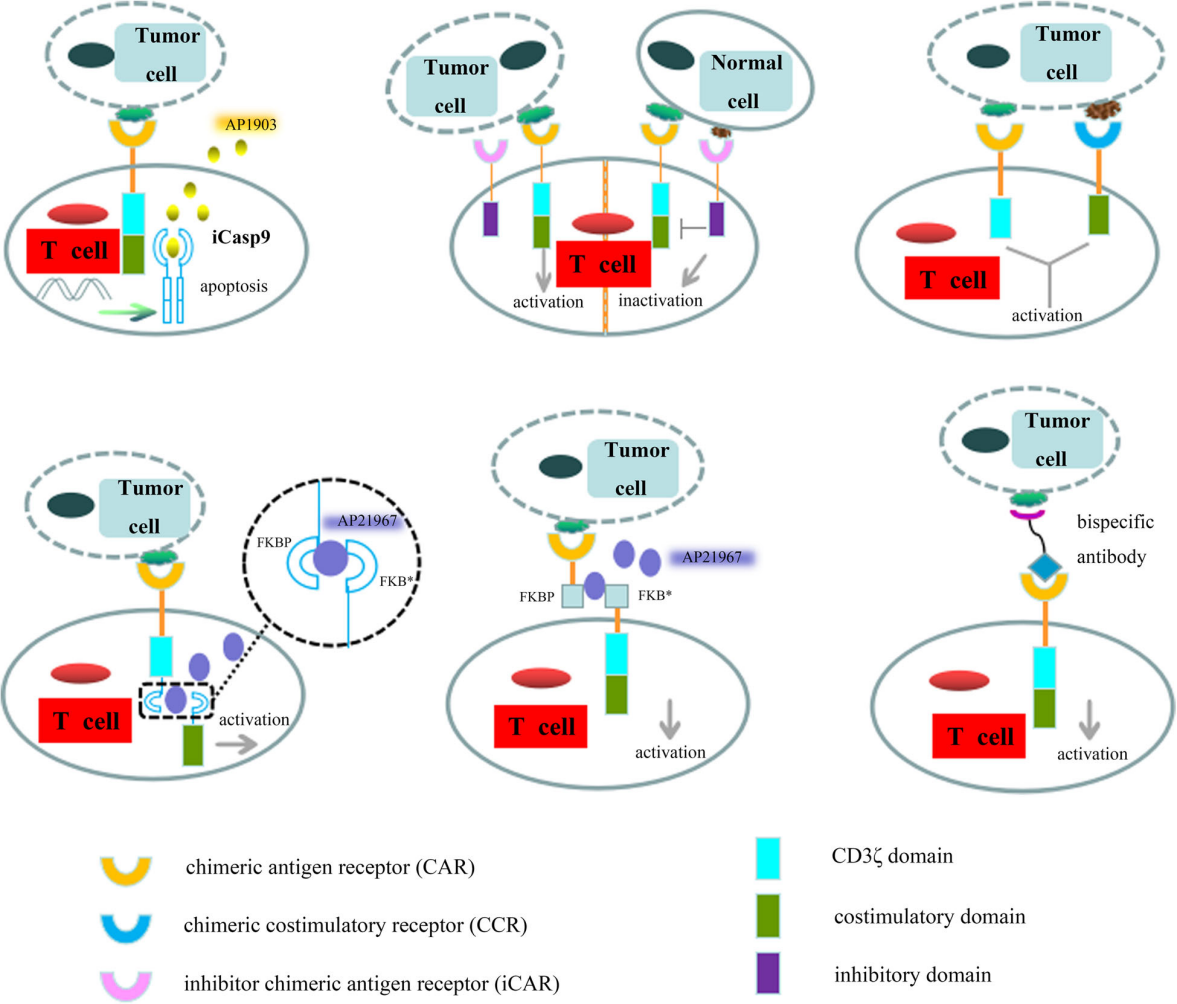
Building strategies to improve the safety of CAR-modified T cells therapy. For upper left, when the release of cytokines by CAR-T cells after killing tumor cells becomes more pronounced than at basic levels, such as interleukin-2 (IL-2) and interferon-γ (IFN-γ), the inducible caspase 9 (iCasp9) can be dimerized, which usually leads to the rapid apoptosis of T cells expressing the iCasp9 suicide gene by addition of a synthetic dimerizing drug AP1903. For upper middle, to achieve the precise regulation of the CAR-T cells, the inhibitory strategy usually harnesses an inhibitory receptor structure comprising an antigen recognition domain with specificity to antigens only presented on normal tissue, and an inhibitory signaling domain to abort T cell behavior despite concurrent engagement of an activating receptor. In this way, the iCAR-T cell can distinguish the tumor and off-tumor cells, and reversibly restrict CAR-T cell functions in an antigen-selective manner, such as the PD-1- and CTLA-4-based iCARs. For upper right, the modified-T cell cotransduced with a CAR, which stimulates an activation signaling pathway upon binding to the first antigen and a chimeric costimulatory receptor (CCR) that recognizes a second antigen to provide the costimulatory signal, can eliminate the cancer cells expressing both antigens rather than either antigen alone. This dual receptor pattern provides a safe path to restricting the activity of engineered T cell in vitro and in vivo. For lower left and lower middle, the split CAR-T cell with two physically separate ports exert the therapeutic functions in the presence of tumor antigens and a heterodimerizing small molecule, AP21967. For lower right, bispecific antibodies are used as a switch to control the interaction between the cancer cell and CAR-T cell. The lower three strategies are similar, in which the cytotoxicity of CAR-T cell are dependent upon presence of exogenous molecule and tumor antigens

**Fig. 3:**
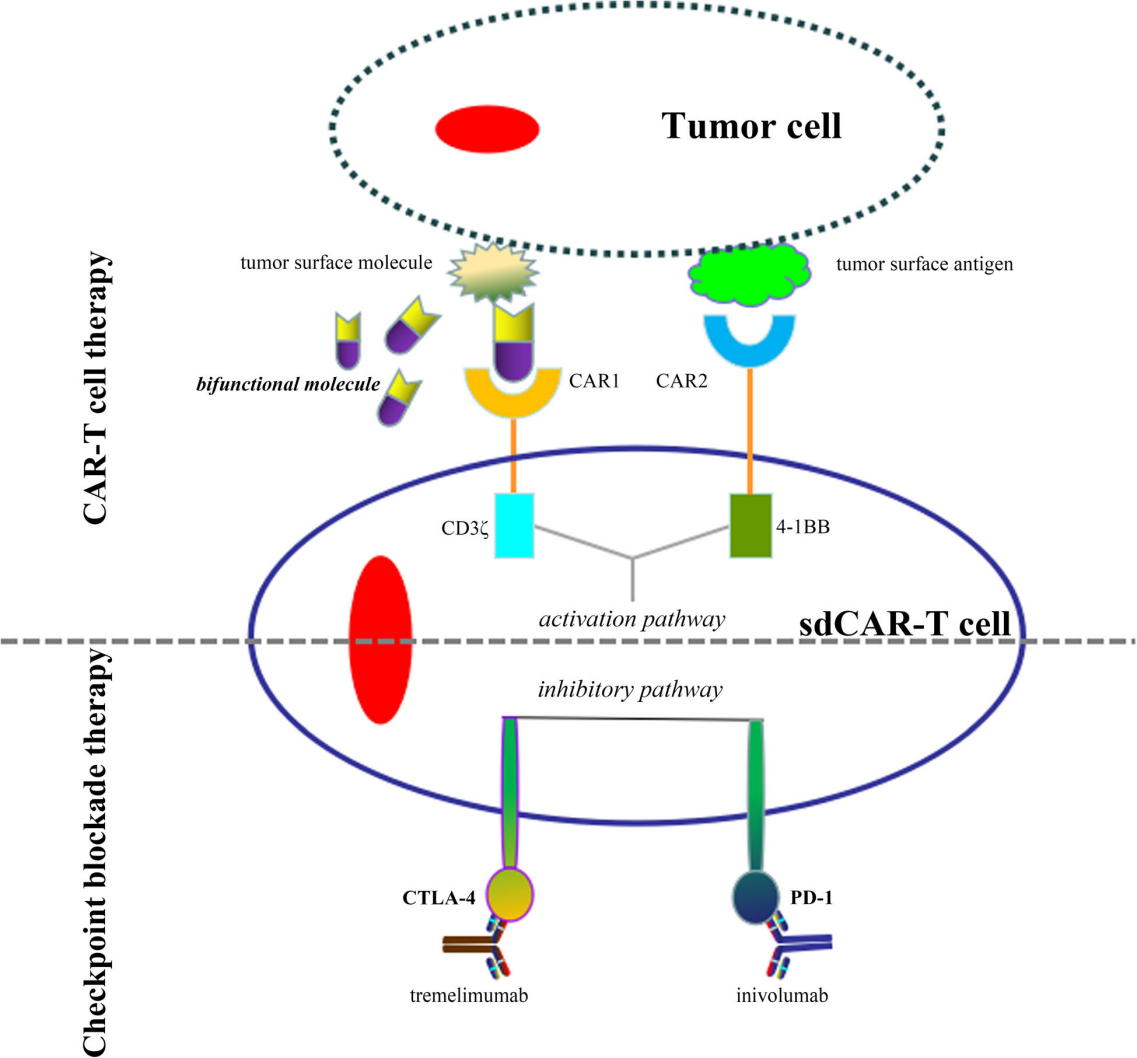
Combinatorial strategy for therapeutic T cell that integrates activation signaling pathway and inhibitory signaling pathway. In the promotion of the activation signaling pathway, this design conformation of the modified-T cell can yield safer therapeutic function upon the safe platform, including the dual receptor engineering T cell and bifunctional molecules. In this system, the T cell that carry two receptors can engage the molecules and tumor surface antigens, and the bifunctional molecule can target T cells and cancer cells. On the other hand, blocking the inhibitory signaling pathway by adding some monoclonal antibodies, such as tremelimumab and nivolumab with specificity for CTLA-4 and PD-1, respectively, was also found to improve the efficacy and persistence of the infused CAR-T cell in vitro and in vivo

During the past few years, many modulations of the CAR-T cell have been developed and proved to be effective and safe [62–64]. In this review, the characteristics of these new technologies are summarized and analyzed. Based on previous research into technology to improve the safety of CAR-T cells (Fig. 2), the autonomous control (e.g., homing and specific recognition of the CAR-T cell) is the first-choice way of ameliorating T cell security. This involves autocrine cytokines against the tumor immunosuppressive microenvironment and the multireceptor presented on the T cells surface that specifically reject the cancer tissue with some tumor associated antigens. Later, some synthetic control devices that can alter the CAR-T cell activity can be implemented in some studies combined with exogenous molecules, which can produce *“smart T cell”* whose therapeutic functions are precisely controlled over the timing and dosage, thereby alleviating toxicity. These strategies offer specific advantages, and the combinatorial strategy involving the dual-receptor T cell and a dependent-molecule may become increasingly viable. In this system, the effective CAR-T cells can target the cancer cell attributed to the receptor with a specificity for the tumor antigen, and then be activated over the exogenous molecules targeting the other receptor (Fig. 3). Additionally, the receptor recognition domain of controlled molecules was coupled to another molecule carrying antitumor activity as a way to achieve higher cytotoxicity.

### Strategies to improve the safety and efficacy of CAR modified-T cells

#### Suicide gene

A safe and efficient means of resolving these adverse effects is the incorporation of suicide genes into the engineered T cells. In this approach, a molecule inciting the cells to apoptosis is administered in an adverse event for killing the transduced-T cell with a suicide-gene product. Based on the previous reports, there are, to date, two types of the suicide genes that have been integrated into CAR-T cells. Initial research into this field showed the herpes simplex virus thymidine kinase (HSV-TK) to be expressed in donor T cells, which has shown noticeable function as a safety switch in clinical trials for cellular therapies [65, 66]. The cell death caused by this suicide strategy, however, may take a long time and remove some of the engineered T cells resulting from the functional mechanism related to blockage of the DNA synthesis [67, 68]. Nevertheless, because it is derived from the virus, HSV-TK may be immunogenic in humans [69].

With the advance in suicide-gene technology, a suicide system based on an inducible caspase-9 (iCasp9) protein was activated through a specific chemical molecule, which has been shown to be safe in vivo [70]. This *“safety switch”* can be inveterately and efficiently expressed in human T lymphocytes and facilitate the maintenance of natural phenotype and antigen specificity [71–73]. In this suicide system, the suicide gene is composed of the sequence of the FK506-binding protein with an F36V mutation (FKBP12-F36V) that has a high affinity to a small-molecule, AP1903, and a gene encoding human caspase 9 switch (Fig. 2 upper left) [73]. Experiments show that iCasp9 suicide gene can induce approximately 99% of transduced cells apoptosis in vitro and in vivo with a 10 nM dose of AP1903 [73]. The iCasp9-based cell has some potential advantages over the HSV-TK suicide gene for cellular therapy [74]. For instance, the iCasp9 system is humanized through the involvement of a human gene, resulting in the low potential immunogenicity. Furthermore, administration of the exogenous AP1903 has an effective and specific ability to eliminate the cell with a transgene expression rather than untransduced cells. A novel 4^th^ generation CD30 CAR-T cell engineered with a self-withdrawal mechanism (FKBP-iCasp9) has shown both efficacy and safety in lymphoma patients in clinical trial NCT02274584. Another clinical case on iCasp9 suicide switch, NCT02414269, the purpose of this phase I study is to test the safety of different doses of the mesothelin-targeted CAR-T cell in patients with malignant pleural disease. Although this method is an important part of the toolbox for engineering therapeutic T cells, it has several intrinsic defects [75, 76]. The suicide switches thoroughly abort the activity of infused cell with a complex and expensive manufacture in an irreversible fashion. In addition, the suicide gene in some modified cells may not act quickly enough to eliminate the off-tumor toxicity during initial cell transfer in vivo because the death of target cells may take several minutes after drug administration.

#### Inhibitory chimeric antigen receptor

An effective strategy for the moderation of immunotoxicity derived from the therapeutic cells depends upon the use of exogenous inhibitors that possess some cytostatic or cytotoxic effects for CAR-T cells, such as corticosteroids [77]. However, this user control approach fails to discriminate between beneficial and deleterious T cell functions in some clinical trials. Additionally, exogenous drugs may trigger several other adverse effects, especially severe organ damage [78]. Next, the negative regulatory receptors engineering strategies, such as PD-1 and CTLA-4 immune inhibitor receptors, which can mitigate the cytotoxicity responses of CAR-T cell when a specific *“protect me”* ligand presented only on the healthy tissue is recognized and captured (Fig. 2 upper middle) [79]. In general, the inhibitory receptors can be upregulated to regulate the number of activated T cell when the immune responses were activated. Based on the mechanism of immune inhibitory receptors, Fedorov et al. described an inhibitory chimeric antigen receptor (iCAR) that can override the T cell responses, and results demonstrated that the iCARs can specifically hinder T cell behaviors from activation, proliferation, and cytokine secretion via the surface TCR or CAR domain with targeting tumor antigen [3, 79, 80].

To prevent the unwanted effects of T cell therapy, the iCAR-engineered T cell can selectively produce cytotoxicity only when the activating receptor comes into contact with tumor antigen, and then transition to a resting state when the inhibitory receptor is targeted with the antigens that are only presented on normal tissues. Notably, this method is a powerful brake for T cells because the inhibitory receptors specifically expressed on activated T cell are mobilized to effectively limit T cell responses. Generally, this genetically engineered receptor regulates T cell responses in an antigen-selective manner. However, unlike antibody-mediated checkpoint blockade, the iCAR-T cells cannot control their spatio-temporal activity.

#### Dual-antigen receptor

Another means of increasing the safety of engineered T cell can be demonstrated upon coexpression of two antigen receptors to target two different tumor-associated antigens (Fig. 2 upper right) [81]. Because of the rarity of real tumor specific antigens, to date, some engineering strategies that can specifically identify a tumor-specific antigen through an engineered CAR or TCR are restricted to apply for killing tumor, especially in solid-tumor cancers. Therefore, the dual-antigen receptor of engineered T cell module has been reported to have less intense side effects even in the absence of a truly tumor-restricted antigen. In previous studies, two types of this module have been characterized in combinatorial recognition manner [82, 83]. Here, we make a conclusion about their advantages and describe the different mechanisms of the killing pathway. First, based on the mechanism of antigen encounter and the stage of T cell activation, the dual pathway from the interaction between T cell and antigen-presenting cell is required for full activation of the T cells [84]. The first signal pathway involves tumor antigen recognition through the extracellular TCR complex and the activation signal transduction with the CD3ζ domain. If the engineered T cells have only the first signal upon the TCR-CD3ζ, however, the activation and durability of T cell are usually transient, such as the first generation of CAR-T cell. The complementary signal pathway provided by costimulatory molecules on antigen presenting cells promotes the survival and expansion of the modified-T cell. The split module of signal pathway helps the T cell control tumor accurately as a result of the dual targeting approach [82, 85]. In 2012, Kloss and coworkers presented a tumor-sensing approach to successfully redirect the T cell specificity for a tumor tissue in the absence of a truly tumor-specific antigen. In this way, the dual-receptor T cell can secrete cytokines and exhibit cytotoxicity once the CAR encounters the first antigen and a chimeric costimulatory receptor (CCR) targeting another antigen [82]. To investigate the antitumor activity of this engineered-T cell for prostate tumors expressing prostate stem cell antigen (PSCA), prostate-specific membrane antigen (PSMA) or both antigens in vivo, they intravenously injected the dual-receptor T cell showing CAR and CCR with a specificity for PSCA and PSMA, respectively, and then tested only for the robust proliferation and tumor eradication in mice bearing the double-positive tumors. The treatment resulted in complete long-term survival rather than in single-positive tissues. However, this tumor-sensing method may have a potential “on-target, off-tumor” effect due to the modified-T cell activated by dual-target tumor can eliminate the normal tissue expressing single tumor-associated antigen specificity for the CAR [86].

Synthetic Notch (synNotch) receptors were developed by Roybal et al. to serve as a general platform for generating novel cell-cell communications toward the purpose of producing a safer and more effective dual-receptor T cell. Results were made public in two recent reports in *Cell* [83, 87]. In this system, activating T cells requires two mechanical processes. First, the synNotch receptor can release a transcription factor to control a CAR expression when the T cell recognizes the first antigen, the tissue-specific antigen. Secondly, the CAR targeting the second antigen causes the T cell to enter a state in which the effect cell has a high activation, proliferation, and cytotoxicity for target cells. On account of the SynNotch receptor to gate and confine the expression of the CARs, it increases the landscape of targetable antigens for CARs and simultaneously reduces the toxicity derived from the use of conventional CARs [88]. This makes this is a unique way in which the synNotch receptors and CARs could improve the therapeutic ability of T cell to identify tumor sites with a high specificity and accuracy.

#### ON-switch CAR

With the advance of strategies for controlling the timing and intensity of engineered T cells, Wu and colleagues recently proposed a complementary positive-regulation manner that the therapeutic cell, termed an ON-switch CAR-T cell, can eliminate target cells bearing cognate antigens in the presence of an exogenous molecule, rapamycin analog AP21967 (Fig. 2 lower left) [89]. Unlike other positive regulations, this ON-switch structure can target the tumor tissue attributed to the CAR with a specificity to tumor antigens, facilitating the gradual titration of therapeutic activity to appropriate levels by varying the concentration of the molecules, and regulate the timing of T cell activation through addition or removal of the small molecule in order to mitigate some severe toxicities. They have constructed the ON-switch CAR with split synthetic receptor system in which the first part of the receptor mainly contains an antigen binding domain, scFv, and another part features two downstream signaling elements, CD3ζ and 4-1BB. In this way, the immunoreactivity of therapeutic cell depends upon the tumor antigen and a small molecule.

Similar to this design, Juillerat et al. describe a method to construct *“transient”* CAR-T cells with a new CAR architecture that is directly dimerized at the hinge domain with addition of the specific molecule (Fig. 2 lower middle) [90]. Finally, they confirmed that the convenient-to-operate strategy mentioned in their report can offer a basic platform to use alternative split-CARs and show a safer way toward the development of the engineered CAR-T cell. In summary, the type of exogenous control behavior based on small molecules here relates to the general principle of integrating autonomous signals with input control. The ON-switch CAR and transient CAR can be implemented for the modified-T cell resulting in ultimately altering conventional T cells into smart T cells whose therapeutic behaviors are precise and effective and subject to user control.

#### Bifunctional molecules as switches

With the rapid development of the bispecific antibodies in cancer therapy, using the bifunctional molecule as a switch to control the activity of T lymphocytes is another exogenous approach to enhance the safety and efficacy of infused immune cells. In the field of immune cell therapy, the bispecific antibodies can be developed as an efficacious bridge to recruit the cytotoxic T cells to kill cancer cells while simultaneously targeting CD3 molecules of T cell and tumor-associated antigen presented on cancer cell surface, resulting in T cell activation and then the destruction of the target cell (Fig. 2 lower right) [91]. This novel design may ultimately overcome some hurdles for the safety of current CAR-T cell immunotherapy and provide a promising approach to improve treatment effects. The results of research performed by Sun et al. show that the anti-CD19/CD3 bispecific T cell engager (BiTE) has had some encouraging clinical effects [92]. Then the anti-CD20/CD3 BiTE was also constructed using *“knobs-into-holes”* technique. Some efficacy studies in murine models demonstrate that the anti-CD20/CD3 molecule can kill B cells and show a high specificity for both effector and target cells even at low doses. The principal mechanistic features of the anti-CD20/CD3 described in their report involve broad activity against malignant B cells with very low CD20 expression levels, and the cytotoxicity for target cell through the granzyme and perforin pathway of T cell. Kim and collaborators have provided another form of bifunctional molecule, consisting of folate coupled with fluorescein isothiocyanate (folate-FITC), which can redirect and regulate the activity of the FITC-specific CAR-T cells toward tumor cells with folate receptors (FR) [93]. In their experiment, this switchable platform has shown a high specificity for FR-positive cells with no activity against FR-negative cells, which demonstrates the specific redirection of the CAR-T cell by folate-FITC molecule. Finally, they confirmed that the cytotoxicity of the modified-T cell is strictly dependent on the presence of both folate-FITC molecule and FR-positive cells, and the CAR-T cells eventually eliminate the target cells with this bifunctional molecule in a concentration-dependent manner.

The latest research papers on a peptide-specific switchable CAR-T (sCAR-T) published in *PNAS* have depicted a new engineered-T system which the sCAR-T cell, switch and target cell can assemble in a spatio-temporal control manner [94, 95]. In vivo in case of B-cell leukemia, the activation, tissue-homing, and cytokine release of sCAR-T cells can be controlled upon administration of small switches consisting of a peptide neo-epitope (PNE), and a scFv specificity for target antigen, such as PNE-CD19 and PNE-CD22. The ability of another universal CAR-T, called anti-FITC CAR-T cell, was confirmed using the FITC-CD19 molecule or FITC-CD20 molecule in xenograft models, resulting in potent, dose-dependent antitumor activity, and slight toxicity. In conclusion, the versatile strategy related to many different tumor antigens with a single-scFv CAR-T cell should be an effective means of surmounting the tumor escape variants and heterogeneity, and can also simplify manufacturing of engineered-T cells for different tumor antigens, which greatly reduces expense [96].

## Conclusions and perspectives

Through some clinical trials on the impressive activity of the modified-T cells, such as ClinicalTrials.gov nos. NCT01029366, NCT02030847, and NCT02388828, this cell-based therapy has been given high expectations for tumor eradication. Even though successful tumor eradication, this form of immunotherapy can cause some systemic life-threatening adverse effects, such as CRS and “on-target, off-tumor” toxicity. In some previous reports, strategies toward amelioration of the adverse effects derived from the CAR-T cell therapy in patients with some serious diseases can be divided into two categories. First, some forms of the CAR structure and autocrime cytokines have been developed to enhance the CAR-T cell abilities for its specific recognition and homing. On the other hand, the timing, dose, and location of the CAR-T cell therapy can be precisely regulated in vitro and in vivo upon addition of some molecules that are used as a switch and rheostat to achieve a remote control for therapeutic T cells.

In this review, the forms of cellular control represent autonomous control (e.g., tumor antigens), user control (e.g., small molecules), or both. This strategy toward improving the safety of CAR-T cell therapy involves the use of active molecules, including dimerization molecules and bifunctional molecules. This exogenous approach to cellular regulation is likely to become increasingly safe and effective, and also allows for more precise control over the timing and location of the immune response. With advances in the dual-receptor paradigm in the field of cell therapy, the new mode of synthetic combinatorial system that includes the dual-receptor CAR with the bifunctional molecules comprising targeted molecule and antitumor molecule may provide an important platform for producing more controllable cellular therapeutic T cells (Fig. 3). The switchable dual-receptor CAR-T characterized above, termed sdCAR-T, may have some advantages with bifunctional molecule for antitumor effect. Firstly, the immune response of sdCAR-T cell against the tumor tissue is dependent on the bifunctional molecule as a switch to simultaneously redirect the tumor cell and molecule-specific CAR-T cell. Second, the therapeutic activity of the dual-receptor CAR-T cell can be titratable by varying concentration of the bifunctional molecule, resulting in precision-control of the therapeutic T cell. Third, the durability of the CAR-T therapeutic effects is greatly increased due to the bifunctional molecules with a long half-life in vivo and in vitro relative to the constitutive single-molecule. Finally, the overall antitumor activity of this combinatorial system will be improved associated with the exogenous molecule carrying the ability to suppress tumor growth. In this way, the combinatorial therapy, a promising pattern of the cell therapy in the war against cancer, now raises the possibility that the tumor cells could be killed based on autonomous tumor antigens and active molecules. This method may have better safety and efficacy than current CAR-T cell.

Conclusively, the cancer biotherapy, including the adoptive immunotherapy with genetically modified T cells and immune checkpoint blockade therapy, has produced better antitumor response than other therapies. Integration therapy involving modified-T cells and immune checkpoint blockade may be an effective means of ultimately eliminating tumor cells (Fig. 3). The switchable CAR-T cells have a controllable cytotoxicity for mitigating tumor burden with the bifunctional molecules. Additionally, using monoclonal antibodies to target the immune checkpoints may produce more efficient T cell behavior. In this way, this combinatorial control system may provide a valuable insight for further refining spatio-temporal control of CAR-T cell therapy.

## Abbreviations

ACT: Adoptive cells transfer
BiTE: Bispecific T cell engager
CAIX: Carboxyanhydrase-IX
CAR: Chimeric antigen receptor
CCR: Chimeric costimulatory receptor
CRS: Cytokine release syndrome
CTC: Common toxicity criteria
CTLA-4: Cytotoxic T lymphocyte-associated antigen 4
ECM: Extracellular matrix
FITC: Fluorescein isothiocyanate
FKBP12-F36V: FK506-binding protein with an F36V mutation
FR: Folate receptor
GvHD: Graft-versus-host disease
HPSE: Heparanase
HSV-TK: Herpes simplex virus thymidine kinase
iCasp9: Inducible caspase-9
IFN-γ: Interferon γ
IL-12: Interleukin 12
IL-6: Interleukin 6
ITAMs: Immunoreceptor tyrosine activation motifs
MHC: Major histocompatibility complex
PD-1: Programmed death 1
PNE: Peptide neo-epitope
PSCA: Prostate stem cell antigen
PSMA: Prostate-specific membrane antigen
sCAR: Switchable CAR
scFv: Single-chain antibody fragments
sdCAR: Switchable dual-receptor CAR
synNotch: Synthetic notch
TCR: T cell receptor
